# Temel Y, Leentjens AFG, de Bie RMA, Chabardes S, Fasano A, editors. Fundamentals and clinics of deep brain stimulation: an interdisciplinary approach: Springer Nature Switzerland AG; 2020. **ISBN 978-3-030-36345-1 ISBN 978-3-030-36346-8 (eBook); https://doi.org/10.1007/978-3-030-36346-8**

**DOI:** 10.3325/cmj.2021.62.104

**Published:** 2021-02

**Field of medicine:** neurosurgery, neurology, clinical neuroscience, psychiatry, medical education.

**Audience:** Neurosurgeons, neurologists, psychiatrists, neuroscientists, senior residents engaged in the clinics of deep brain stimulation, and other interested physicians.

**Purpose:** The book provides an overview of the basic knowledge on the clinics of deep brain stimulation (DBS), including anatomy and neurophysiology of the targets, surgical and technical aspects, programming, patients care, ethical consideration, as well as of neurological and psychiatric conditions treated by DBS. The book provides a wealth of information and can serve as a useful resource for all interested readers.

**Content:** The content is divided into three parts: *General Section, Neurology,* and *Psychiatry.* The first part begins with a chapter providing a historical perspective of the neurosurgical treatment of psychiatric and movement disorders in the first half of the 20th century, leading to the introduction of stereotactic neurosurgery in clinical practice (Spiegel and Wycis, 1947). Furthermore, it describes levodopa and stereotactic neurosurgical procedure periods in the second half of the 20th century. A new era in the history of DBS started in 1987, since when DBS has made several significant breakthroughs in the treatment of different conditions.

This chapter is followed by a chapter focusing on the organization of the cortico-basal ganglia-thalamocortical circuits, and providing anatomical details of the structures important for DBS such as the striatum, subthalamic nucleus, globus pallidus, and thalamus.

The functioning of DBS is covered in the third chapter. This chapter presents the hypothesis on the potential mechanisms of high-frequency stimulation and the effect of DBS on target nuclei, downstream nuclei (exciting afferent and efferent projections), and cortical neuronal activity. It also describes the effects of subthalamic nuclei high-frequency stimulation on the modulation of dopamine neurotransmission in Parkinson’s disease (PD).

The fourth chapter, *Surgical and Technical Aspects of Deep Brain Stimulation,* deals with the stereotactic implantation technique of DBS electrodes, usage of different stereotactic atlases, stereotactic frame-based navigation, intraoperative neurophysiological and clinical testing, intraoperative imaging for lead verification, and steering and adaption of stimulation. It also describes alternative implantation techniques, such as Nexframe and robot-guided surgery, as well as delineates future directions in technical developments of imaging using diffusion tensor imaging, connectivity, and functional magnetic resonance.

The following chapter is dedicated to adaptive DBS – the general concept and principles, as well as its clinical and technical implementation. Namely, it deals with the systems based on a closed-loop system analyzing the variables that reflect the patients’ clinical state and determining whether stimulation parameters need adjustment. Adaptive DBS in experimental clinical settings is investigated in several clinical conditions, such as PD, essential tremor, dystonia, epilepsy, Tourette’s syndrome, and obsessive-compulsive disorder.

The sixth chapter briefly describes the neurophysiology of the basal ganglia as illustrated in PD. It focuses on neurophysiological models of the basal ganglia, such as the classical direct/indirect model, reinforcement learning model, and multi-objective optimization model, as well as the neurophysiology of DBS.

In the seventh chapter, *Anaesthesia for Deep Brain Stimulation Surgery,* preoperative considerations and investigations for specific conditions (PD, essential tremor, dystonia, epilepsy, obsessive-compulsive disorder, Tourette's syndrome) are presented, with a special emphasis on anticoagulants and antiplatelet therapy during DBS. Various anesthetic techniques for DBS procedure are described: monitored anesthesia care with local or regional anesthesia, conscious sedation, asleep-awake-asleep technique, and general anesthesia, as well as potential perioperative complications and postoperative management.

The eighth chapter briefly describes a variety of approaches to DBS programming, discussing measured proximate effects of DBS and voltage-gated ionic conductance channels, interaction of DBS and pharmacotherapy, as well as challenges in patients programming (constant current vs constant voltage, selectivity of action potentials, generation, amplitude, frequency, electrode configurations and their special features, etc).

The impact of DBS on cognition and psychiatric side effects of DBS for patients with movement disorders, as well as on post-operative psychosocial functioning, are described in the ninth chapter. This chapter also deals with cognitive and psychiatric effects of DBS and their prevention and reduction, along with the role of the neuropsychologist in the management of post-operative psychosocial adjustment of patients.

The tenth chapter discusses ethical issues of DBS, such as respect for autonomy, beneficence, and nonmaleficence, justice, research ethics. A special emphasis is placed on ethics in DBS for psychiatric conditions and the gray area of experimental treatments.

The last chapter of the first part deals with DBS patient selection and counseling, multidisciplinary team evaluation, postoperative care, social support, lifestyle issues, and outcome evaluation.

The second part, *Neurology,* is dedicated to neurological disorders treated by DBS: PD, tremor, dystonia, epilepsy, and Gilles de la Tourette syndrome. The chapters focus on pathophysiology, selection for surgery, target selection, surgical approach, results and side effects of DBS, followed by programming and long-term follow-up.

The third part, *Psychiatry,* deals with DBS treatment of psychiatric disorders: obsessive-compulsive disorder, depression, and other indications (addiction, Huntington’s disease, anorexia nervosa, minimally conscious state, Alzheimer’s disease, and schizophrenia). The chapters briefly describe clinical presentation, conventional treatment, and pathophysiology, selection for surgery, surgical approach, therapeutic results, programming, and side effects of DBS in these disorders.

**Highlights:** The book contains all the essential information and provides an excellent overview of the history of stereotactic neurosurgery, basic anatomical and physiological information, DBS mechanisms, surgical techniques and programming, patients' neuropsychological assessment, organization of care, as well as ethical considerations in general. Specific conditions are discussed in separate sections dealing with neurological and psychiatric disorders. The comprehensive index facilitates orientation and selective reading. The text is accompanied by numerous color figures, schematic presentations, neuroradiological images, and photographs, which make the learning easy and exciting.

**Related reading**: A number of other comprehensive medical references, either in book or pocket-size handbook format, are available, such as: Stereotactic and Functional Neurosurgery. Principles and Applications, Springer, 2020; Handbook of Deep Brain Stimulation, Nova Science Publishers, 2020; Deep Brain Stimulation: Techniques and Practices, Thieme, 2019; Deep Brain Stimulation: Indications and Applications, 1st Edition, Taylor & Francis Group, 2017; Deep Brain Stimulation Programming: Mechanisms, Principles and Practice, Oxford University Press, 2016; Deep Brain Stimulation for Neurological Disorders. Theoretical Background and Clinical Application, Springer, 2015; Deep Brain Stimulation Management, 2nd Edition, Cambridge University Press, 2015; Deep Brain Stimulation A New Frontier in Psychiatry, Springer, 2012.

